# Supplementary material for: Splice-Junction-Based Mapping of Alternative Isoforms in the Human Proteome
Source: Cell Rep. Author manuscript; Available in PMC 2020 Jan 15. (PMC6961840; doi:10.1016/j.celrep.2019.11.026)
Supplement: 2 [file NIHMS1546469-supplement-2.zip › DF1/PXD010154/Heart/Heart_58_TPM3_AISEELDHALNDMTSGSQMLKLDK.pdf]

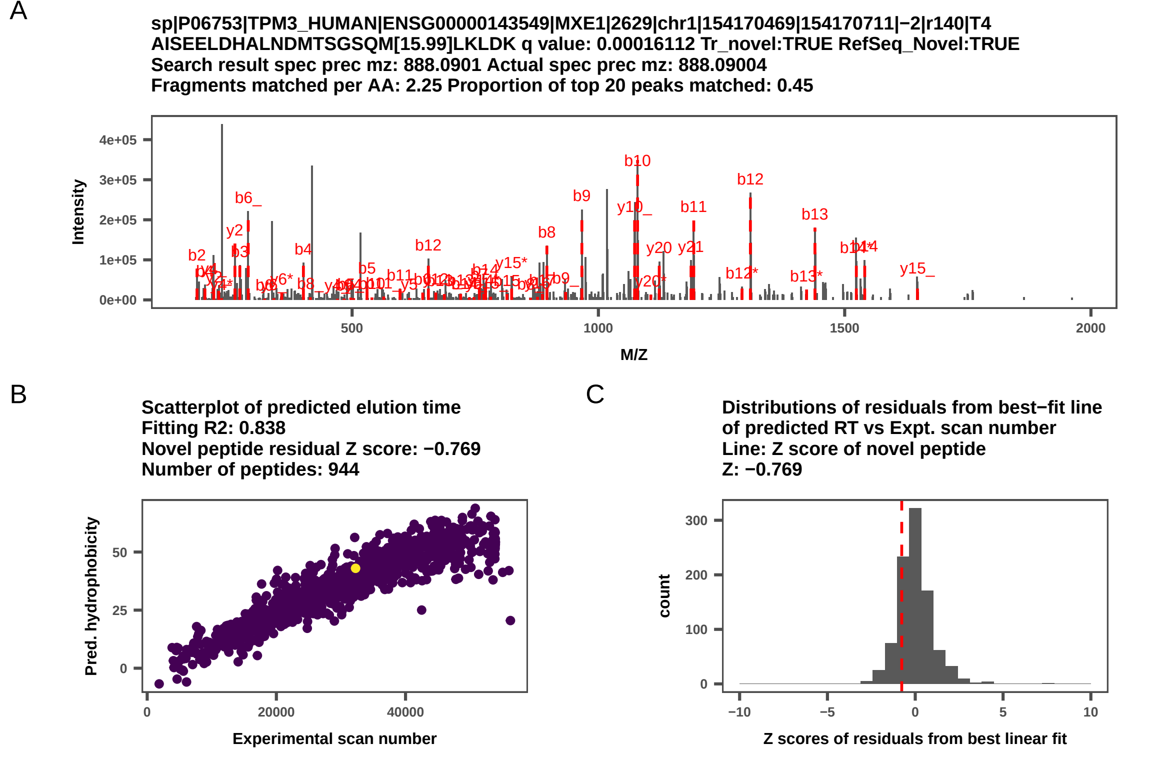
A
sp|P06753|TPM3_HUMAN|ENSG00000143549|MXE1|2629|chr1|154170469|154170711|−2|r140|T4
AISEELDHALNDMTSGSQM[15.99]LKLDK q value: 0.00016112 Tr_novel:TRUE RefSeq_Novel:TRUE
Search result spec prec mz: 888.0901 Actual spec prec mz: 888.09004
Fragments matched per AA: 2.25 Proportion of top 20 peaks matched: 0.45
Intensity
4e+05
3e+05
2e+05
1e+05
0e+00
b10
b12
b9
b11
b13
b6_
y10_
y2
b8
y20
y21
b3
b4
b12
b2
y15*
b14*
b12*
b13*
y15_
b5
y20*
M/Z
500
1000
1500
2000
B
Scatterplot of predicted elution time
Fitting R2: 0.838
Novel peptide residual Z score: −0.769
Number of peptides: 944
Pred. hydrophobicity
50
25
0
0
20000
40000
Experimental scan number
C
Distributions of residuals from best−fit line
of predicted RT vs Expt. scan number
Line: Z score of novel peptide
Z: −0.769
count
300
200
100
0
−10
−5
0
5
10
Z scores of residuals from best linear fit
